# Antibacterial Activity of Phyto-Synthesized Silver Nanoparticles From Dryopteris cristata Against Staphylococcus aureus ATCC 28923 and Escherichia coli ATCC 28922

**DOI:** 10.7759/cureus.70856

**Published:** 2024-10-04

**Authors:** Emmanuel T Bello, Sunday Awe, Muritala I Bale, Ayoola Awosika, Janet M Oladejo, Faith J Olaitan, Jedidiah E Ikibe

**Affiliations:** 1 Department of Science Laboratory Technology (Microbiology Unit), Newland Polytechnic, Ilorin, NGA; 2 Department of Microbiology, Kwara State University, Ilorin, NGA; 3 Department of Microbiology and Parasitology, School of Medicine and Pharmacy, University of Rwanda, Kigali, RWA; 4 College of Medicine, University of Illinois Chicago, Chicago, USA; 5 Department of Medical Microbiology and Parasitology, University of Ilorin Teaching Hospital, Ilorin, NGA; 6 Department of Biological Sciences (Microbiology Unit), Thomas Adewumi University, Oko, NGA; 7 Department of Academic Planning, Ojaja University, Ilorin, NGA

**Keywords:** antimicrobial resistance, dryopteris cristata, escherichia coli, microbial pathogens, nanotechnology, phytochemicals, staphylococcus aureus

## Abstract

Introduction

Nanotechnology has emerged as a vital field, particularly in synthesizing nanoparticles. Silver nanoparticles (AgNPs) are recognized for their strong antimicrobial properties against various pathogens, including *Staphylococcus aureus* and *Escherichia coli*, due to their small size and high surface area. Green synthesis using plant extracts offers an eco-friendly alternative. The rise of multidrug-resistant bacteria underscores the urgent need for new antimicrobial agents. This study investigates the antibacterial activities of *Dryopteris cristata* AgNPs (DC-AgNPs) against *S. aureus* and *E. coli*, employing antimicrobial susceptibility testing (AST), minimum inhibitory concentration (MIC), and minimum bactericidal concentration (MBC) assessments, along with nanoparticle characterization.

Materials and method

The antimicrobial activity ofDC-AgNPs was evaluated using clinical isolates of *E. coli* and *S. aureus*. Bacterial inoculums were standardized to 0.5 MacFarlard (1.5 × 10^8^ CFU/mL) and tested via a modified agar-well diffusion method. The MIC and MBC were determined using broth microdilution and sub-culturing methods, respectively. Characterization of the nanoparticles was conducted using Ultraviolet-visible (UV-Vis) spectroscopy, X-ray diffraction (XRD), Fourier-transform infrared spectroscopy (FTIR), and scanning electron microscopy (SEM).

Results and conclusion

*D. cristata* was identified as the plant used to synthesize AgNPs, confirmed by the University of Ilorin, Nigeria. Phytochemical screening revealed the presence of tannins, flavonoids, glycosides, and phenolics. The AgNPs were synthesized by adding the aqueous extract to silver nitrate, resulting in a color change. Characterization via UV-Vis spectrophotometry confirmed nanoparticle formation. Antimicrobial testing showed that DC-AgNPs effectively inhibited *S. aureus* and *E. coli*, with minimum inhibitory concentrations of 125 μg and 250 μg, respectively, indicating their potential as antimicrobial agents.

## Introduction

Nanotechnology has rapidly emerged as a significant field of scientific research, particularly in the synthesis and application of nanoparticles, which range in size from 1 nm to 100 nm. Among these, silver nanoparticles (AgNPs) have garnered a lot of interest because of their distinct biological, chemical, and physical characteristics-particularly their potent antibacterial action against a range of pathogens, including *Staphylococcus aureus* and *Escherichia coli* [1].

AgNPs are recognized not only for their antibacterial effects [2], but also for their anti-fungal, anti-inflammatory, and anti-viral properties [3-5]. AgNPs are a promising candidate for tackling the expanding problem of infectious diseases because of their wide range of activity. Using plant extracts for the green synthesis of AgNPs has become a viable and affordable substitute for conventional chemical procedures in terms of both cost and environmental impact. This approach utilizes the natural reducing and stabilizing properties of plant compounds, eliminating the need for hazardous chemicals and complex laboratory conditions, thereby making it a more sustainable option for nanoparticle production [1]. Numerous studies have successfully demonstrated the green synthesis of AgNPs using various plant extracts, highlighting the potential of medicinal plants in developing natural antimicrobial agents [6].

The increasing prevalence of multidrug-resistant bacteria, particularly strains like *S. aureus* and *E. coli*, poses significant challenges in treating infections. This rise in resistance is primarily attributed to the indiscriminate use of antibiotics in healthcare, agriculture, and veterinary medicine [7,8]. Infectious diseases are a major health issue and rank as the second leading cause of loss of productive life and death globally [9]. The emergence of multidrug-resistant pathogens makes treatment choices more challenging and emphasizes the pressing need for novel antimicrobial agents with broad-spectrum effectiveness. This study focuses on the antimicrobial activities of *Dryopteris cristata*-derived AgNPs (DC-AgNPs) against *S. aureus* and *E. coli*, aiming to contribute to the development of effective treatments for resistant bacterial infections. By exploring the potential of DC-AgNPs, this research seeks to provide insights into new therapeutic strategies that leverage the unique properties of nanoparticles to combat the growing threat of antibiotic resistance, ultimately improving patient outcomes and public health.

## Materials and methods

This study was conducted at Newland Polytechnic and College of Health Technology, Science Laboratory Technology Department, located in Ilorin, Kwara State, Nigeria.

Identification of plants

The plant used in this research was identified as *Dryopteris cristata* by the Department of Plant Biology, University of Ilorin, Nigeria.

Detection of phytochemicals

Chemical assays were conducted to identify the presence of key phytochemical compounds in the extract.

Test for Tannins

The dried extract (500 mg) was boiled in 20 ml of water and then filtered. A few drops of 0.1% FeCl_3_ were added, and the appearance of a blue-black or brownish-green color indicated the presence of tannins.

Test for Flavonoids

A colorimetric method was used to detect flavonoids [10]. The aqueous filtrate of the extract was mixed with 5 ml of dilute ammonia solution, followed by the addition of concentrated H_2_SO_4_. The appearance of a yellow color indicated the presence of flavonoids, which faded over time. When 100 μL of 1% NH_3_ solution was added to a portion of the filtrate, the yellow color reappeared, further confirming the presence of flavonoids.

Test for Terpenoids

The aqueous extract (5 ml) was mixed with 2 ml of chloroform, and 3 ml of concentrated H_2_SO_4_ was carefully added along the wall of the test tube. The development of a reddish-brown color at the interface indicated the presence of terpenoids [11].

Test for Glycosides

A small portion of the plant extract was dissolved in water, and 1 ml of one-molar (1M) sodium hydroxide solution (NaOH) solution was added. The appearance of a yellow color confirmed the presence of glycosides [12].

Test for Phenolics

A small portion of the plant extract was placed in a test tube, and a few drops of 5% ferric chloride solution were added. The development of a bluish-black or greenish-black color signaled the presence of phenolic compounds [13].

Test for Saponins

A small amount of the plant extract was placed in a test tube, followed by the addition of warm distilled water. The mixture was shaken vigorously for two to three minutes and then allowed to stand for 10-15 minutes. The formation of a stable and persistent foam on the liquid's surface indicated the presence of saponins, with the foam height reflecting the saponin content [14].

Test for Cardiac Glycosides

The aqueous extract (5 ml) was treated with 2 ml of glacial acetic acid containing a drop of ferric chloride (FeCl_3_) solution, followed by the addition of 1 ml of concentrated sulfuric acid (H_2_SO_4_). The formation of a brown ring at the interface, indicating the presence of deoxysugar, confirmed the presence of cardiac glycosides [15].

Synthesis of AgNPs using aqueous extraction

For the synthesis of AgNPs, an aqueous solution of 1 mM silver nitrate (AgNO_3_) was prepared. Initially, 1000 ml of the 1 mM AgNO_3_ solution was mixed with 25, 50, 100, and 200 ml aliquots of *Dryopteris cristata* (DC) aqueous extracts. The reaction took place in 1000 ml volumetric flasks at room temperature, with the solutions vigorously mixed on a magnetic stirrer to optimize the reduction of AgNO_3_ by the plant extracts (testing various concentrations to identify the optimum condition). The optimal combination, identified as 100 ml of DC extract with 1000 ml of 1 mM AgNO_3_ solution, was then used to synthesize DC-AgNPs. The reduction of AgNO_3_ to silver ions (Ag+) was confirmed by the appearance of a dark brown coloration. The DC-AgNPs were collected by centrifugation at 4,000 rpm for 15 minutes at room temperature, and the resulting pellets were dried in an oven at 60°C.

Assessment of antimicrobial activity of DC-AgNPs

Test Bacteria and Inoculum Preparations

The utilized bacterial species were clinical isolates *E. coli* (ATCC 28922) and *S. aureus* (ATCC 28923) from the Department of Medical Microbiology, University of Ilorin Teaching Hospital (UITH), Nigeria. Purified cultures were maintained as stocks on appropriate nutrient media. Respective bacterial inoculums were freshly prepared per usage and standardized to 0.5 MacFarland (0.05 ml of 1% barium chloride (BaCl_2_) and 9.95 ml of 1% H_2_SO_4_).

Antimicrobial Susceptibility Testing

The study engaged a modified agar-well diffusion plate technique for antimicrobial effectiveness testing of the synthesized nanoparticles. All test bacterial species were utilized as fresh bacterial (20±2 hours) and fungal (45±3 hours) cultures; stock bacterial inocula were standardized to 0.5 McFarland corresponding to 1.5 x 10^8^ CFU/mL. Prior to pouring the molten but cool sterile Mueller Hinton agar, well-labeled sterile Petri dishes were seeded with 1 ml of fresh (24 hours) standardized test bacteria. Upon solidification, a 0.5 cm diameter sterile stainless-steel cork borer was used to drill wells in the inoculated solid medium. The wells were filled with respective synthesized nanoparticle dilutions. The experimental sets were performed in triplicates and incubated at recommended bacterial growth conditions (35±2 ˚C) in the incubator. The susceptibility of test microbes to the antimicrobial agents was measured by the diameters of the respective zones of inhibition observed.

Determination of Minimum Inhibitory Concentration (MIC)

The MIC was evaluated using the broth microdilution method. The nanoparticle extracts were diluted two-fold with sterile distilled water to yield 50%, 25%, 12.5%, 6.25%, 3.13%, 1.57%, and 0.78%, the MICs were determined against the test pathogens in 8 mL peptone broth medium. Sterile distilled water and broth medium were used as negative controls, while the test pathogen and antimicrobial agent in the assay medium served as positive controls. A 100 μL inoculum of standardized microbial suspension was added to each medium-containing tube, except the controls, followed by 100 μL of antimicrobial agent. Incubation was at 35±2°C for 20±4 hours accordingly. The turbidity of sets (indicating microbial proliferation) was compared and the tube with the lowest concentration and visible clarity was selected as the MIC. All experimental setups were performed in triplicates. For the determination of minimum bactericidal concentration (MBC), 10 μL from each broth tube that exhibited no turbidity or growth were taken, isolated with nutrient agar, and incubated at 35±2°C for 24 hours. The lowest concentration that revealed no visible bacterial growth after sub-culturing was taken as MBC. Positive and negative cultures were also prepared.

Optimization process for AgNP synthesis and antibiotic sensitivity testing

In the optimization process for synthesizing AgNPs, various concentrations of aqueous extracts from *D. cristata* were tested in combination with a 1 mM AgNO_3_ solution. The objective was to determine the most effective ratio that would facilitate the reduction of Ag+ and promote the formation of AgNPs. After preparing the AgNO_3_ solution, aliquots of the plant extracts were added in varying volumes (25 ml, 50 ml, 100 ml, and 200 ml) to the 1000 ml AgNO_3_ solution. The mixtures were stirred vigorously at room temperature to enhance the interaction between the extracts and the Ag+. Ultimately, the optimal concentration was identified as a 1:10 ratio of aqueous plant extract to AgNO_3_ solution, which yielded the most efficient reduction and formation of nanoparticles, as evidenced by a distinct dark brown coloration indicative of successful AgNP synthesis. Following the synthesis optimization, the 1:10 concentration of aqueous plant extracts to AgNO_3_ was utilized to conduct antibiotic sensitivity tests. This concentration was chosen based on its demonstrated efficacy in producing AgNPs, which are known for their antimicrobial properties. The antibiotic sensitivity tests involved exposing various bacterial strains to the synthesized AgNPs to evaluate their inhibitory effects. The results indicated that the AgNPs synthesized at this optimal concentration exhibited significant antibacterial activity, highlighting the potential of using *D. cristata* not only for nanoparticle synthesis but also for developing effective antimicrobial agents. This dual application underscores the importance of optimizing synthesis conditions to enhance the functional properties of the resulting nanoparticles.

Characterization of nanoparticles synthesized by plant extracts

The standard procedure previously described was followed for the characterization of DC-AgNPs.

Ultraviolet-Visible (UV-Vis) Spectrum Analysis

The preliminary characterization of DC-AgNPs was carried out using a UV-Vis spectrophotometer. The synthesis of AgNPs, achieved through the reduction of Ag+, was monitored by recording the UV-Vis spectra of the solutions at one-hour intervals until no further changes in absorbance were observed. The absorbance spectra of the AgNP solution were measured within the 300-700 nm range, using distilled water for baseline correction.

X-Ray Diffraction Analysis

The AgNPs obtained were characterized in powdered form using an X-ray diffractometer. The diffraction pattern was recorded using CuK_α_ radiation with a nickel monochromator. The average crystalline size of the nanoparticles was calculated using the Scherrer equation: D=Kλβcos⁡θ, where D is the average crystal size of the nanoparticle, λ is the wavelength of the X-ray source (1.54060 Å), β is the width at half of maxima of the diffraction peak, and K is the constant of the Debye-Scherrer equation with the value ranging from 0.9 to 1.0.

Fourier Transform Infrared Spectroscopy (FTIR) Analysis

FTIR is a crucial technique for the identification and characterization of molecules, providing valuable insights into their rotational and vibrational modes. To prepare the DC-AgNPs for FTIR analysis, the nanoparticles were washed three times by centrifugation with deionized water to remove any unbound or loosely attached proteins, enzymes, or other phytocompounds from their surface. The resulting DC-AgNP powder was then diluted with potassium bromide (KBr), and the transmittance spectrum was recorded. FTIR measurements were conducted in diffuse reflectance mode at a resolution of 4 cm⁻¹ using the Spectrum Two FT-IR Spectrometer (PerkinElmer, Inc., Waltham, Massachusetts, United States).

Scanning Electron Microscopy (SEM)

Finely ground DC-AgNP powder was employed for SEM examination. Using an accelerating voltage of 20 kV, the JSM-6510LV scanning electron microscope (JEOL, Ltd, Akishima, Tokyo, Japan) was used to record the images. The elemental composition of DC-AgNPs was analyzed using an SEM equipped with an INCAx-sight EDAX (energy dispersive X-ray analysis) spectrometer (Oxford Instruments plc, Abingdon, United Kingdom).

## Results

Several medicinal plants have been successfully tested and used in synthesizing silver nanoparticles. In this study, the aqueous extract of *D. cristata* was used for the synthesis of AgNPs. *D. cristata* aqueous extracts were added to AgNO_3_ solution, and the change in color indicated a reduction reaction of Ag+ to AgNPs. The characterization of DC-AgNPs is presented through various analytical techniques, revealing critical insights into their composition and potential antimicrobial properties.

Phytochemical screening of the aqueous extract

Table 1 outlines the qualitative phytochemical screening of the aqueous extract, indicating the presence of tannins, flavonoids, glycosides, cardiac glycosides, and phenolics, all of which are known to contribute to antimicrobial activity. The absence of terpenoids and saponins suggests a focused profile of bioactive compounds that may stabilize the nanoparticles and enhance their efficacy.

**Table 1:** Results of phytochemical tests conducted on the the aqueous extract of Dryopteris cristata extract, commonly known as ‘wood crested fern’. The "+" symbol indicates the presence of the respective phytochemicals, while the "-" symbol indicates absence

Aqueous Plant Extract	Phytochemical Tests (qualitative)	Result
*Dryopteris cristata* (Fern plant)	Tanin	+
	Flavonoids	+
	Glycoside	+
	Cardiac Glycoside	+
	Terpenoid	-
	Saponin	-
	Phenolics	+

Characterization of AgNPs

UV-Vis Spectrophotometry

Table 2 presents the results of the UV-Vis spectrophotometric analysis conducted on AgNPs synthesized using the aqueous extract of the crested wood fern plant, *D. cristata*. The absorbance of the reaction mixture was measured at different time intervals ranging from zero to four hours to monitor the formation and stability of the AgNPs. The absorbance values are indicative of the surface plasmon resonance (SPR) of the AgNPs, which is a characteristic optical property arising from the collective oscillation of conduction electrons in response to incident light. The consistent increase in absorbance at specific wavelengths reflects the SPR characteristic of AgNPs, confirming successful synthesis.

**Table 2:** Results of the ultraviolet-visible spectrophotometric analysis conducted on AgNPs synthesized using the aqueous extract of the crested fern plant, Dryopteris cristata The absorbance of the reaction mixture was measured at different time intervals ranging from 0-4 hours to monitor the formation and stability of the AgNPs. The absorbance values are indicative of the surface plasmon resonance of the AgNPs, which is a characteristic optical property arising from the collective oscillation of conduction electrons in response to incident light. AgNP: silver nanoparticle

Wavelength (nm)	0 hours	1 hour	2 hours	3 hours	4 hours
300	2.292	2.367	2.319	2.319	2.319
350	2.022	2.092	2.066	2.056	2.060
400	1.270	1.309	1.316	1.333	1.384
450	1.020	1.040	1.055	1.068	1.110
500	0.685	0.702	0.711	0.721	0.758
550	0.710	0.712	0.711	0.717	0.747
600	0.625	0.620	0.617	0.616	0.648
650	0.445	0.442	0.438	0.437	0.467
700	0.462	0.451	0.444	0.442	0.471

X-ray diffraction and FTIR

**Figure 1:**
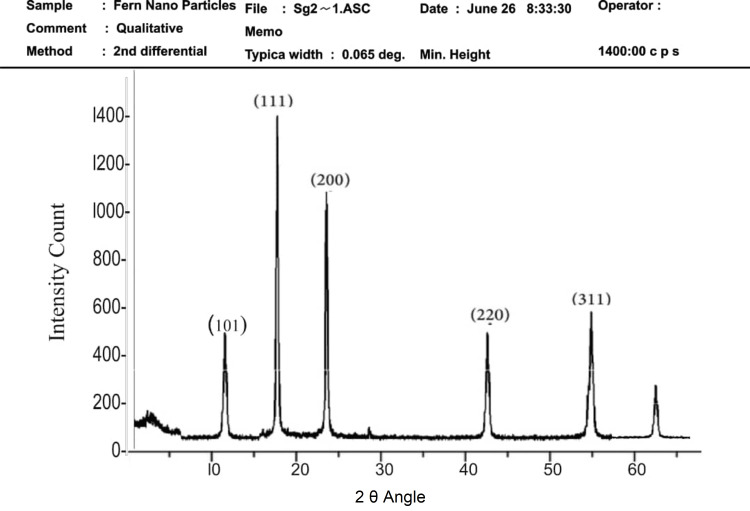
X-ray diffraction analysis of silver nanoparticles synthesized from Dryopteris cristata Sharp peaks are seen at 101, 111, 200, 220, and 311, confirming their face-centered cubic (FCC) crystalline structure with high crystallinity and uniform particle size, as evidenced by the narrow peak width of 0.065 degrees.

**Figure 2:**
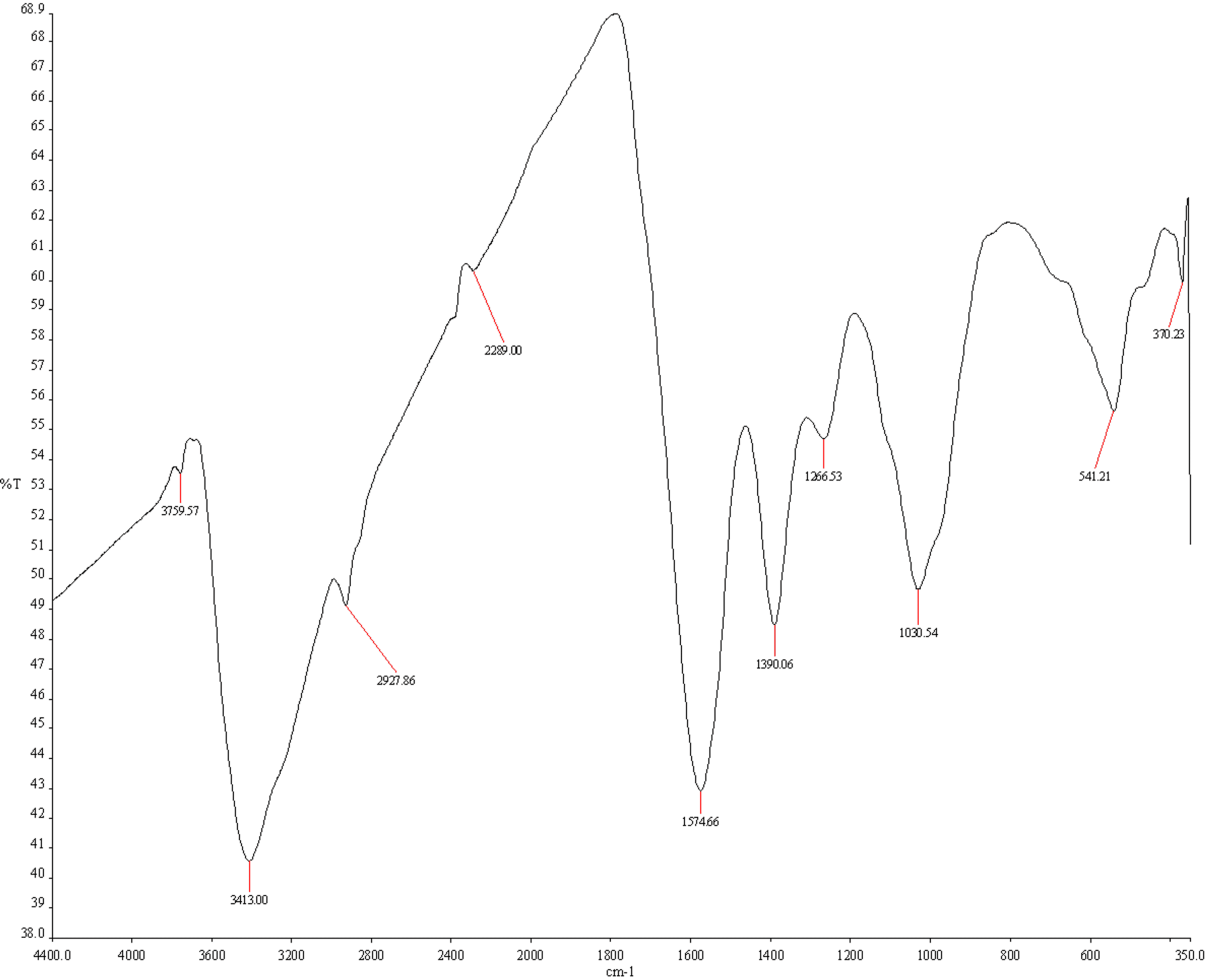
FTIR spectrum of silver nanoparticles synthesized using Dryopteris cristata %T= Percent Transmittance; cm⁻¹= (Wavenumber) FTIR: Fourier-transform infrared spectroscopy

Figure 1, which illustrates the X-ray diffraction pattern, confirms the face-centered cubic (FCC) crystalline structure of the nanoparticles with high crystallinity and uniform particle size. Finally, Figure 2 displays the FTIR spectrum, highlighting functional groups such as hydroxyl and aromatic compounds that may enhance the antimicrobial properties of the nanoparticles by facilitating interactions with bacterial membranes. Collectively, these results suggest that the synthesized DC-AgNPs possess a robust profile of phytochemicals and structural characteristics conducive to antimicrobial activity.

Antibacterial activity of DC-AgNPs

The antibacterial activity of DC-AgNPs was modest, as shown in Table 3, where they exhibited an average inhibition zone of 7.17 ± 0.94 mm against *S. aureus* and no measurable activity against *E. coli*. This limited effectiveness is primarily attributed to the relatively large size of the nanoparticles (approximately 123.4 nm), which correlates with reduced antimicrobial properties. Smaller AgNPs (typically less than 15 nm) demonstrate significantly enhanced antibacterial activity due to their increased surface area and ability to penetrate bacterial membranes more effectively. The larger size of the DC-AgNPs may hinder their capacity to generate reactive oxygen species (ROS) or interact sufficiently with bacterial cells, leading to resistance, particularly in gram-negative bacteria like *E. coli*.

**Table 3:** Antimicrobial sensitivity test for silver nanoparticles synthesized from Dryopteris cristata, showing the average inhibition zones (in millimeters) The results show the effectiveness against *Staphylococcus aureus*. Inhibition zones are measured in millimeters, indicating the varying levels of antimicrobial activity exhibited by the nanoparticles.

Antimicrobial Agent	Staphylococcus aureus	Escherichia coli
*Dryopteris cristata silver nanoparticles* (DC-AgNPs)	7.17±0.94	0
*Dryopteris cristata* aqeous extract	0	0

In Table 4, conventional antibiotics such as sparfloxacin displayed notable inhibition zones against both bacterial strains, emphasizing the need for improved formulations of DC-AgNPs rather than suggesting that conventional drugs are inherently superior. The findings indicate a clear necessity for further research aimed at reducing nanoparticle size or modifying synthesis methods to enhance their antimicrobial efficacy. By optimizing these parameters, it may be possible to develop more effective DC-AgNPs that can overcome resistance and serve as viable alternatives or complementary treatments against bacterial infections.

**Table 4:** Antibiotic activities of conventional antibiotics against test organisms (zones of inhibition measured in millimeters)

Antibiotics	Staphylococcus aureus	*Escherichia coli*
Sparfloxacin	18.5±0.5	5±1.41
Augmentin	4±0	0

The results in Table 5 show the MIC and MBC of *D. cristata* nanoparticles and aqueous extracts against *S. aureus* and *E. coli*. The DC-AgNPs effectively inhibited *S. aureus* at concentrations of 125 μg and 250 μg, indicating their potential as an antimicrobial agent against this gram-positive bacterium. However, *E. coli* exhibited resistance to the aqueous extracts, as well as the DC-AgNPs with MIC values exceeding 250 μg, highlighting the need for further optimization of DC-AgNPs to enhance their efficacy against resistant gram-negative bacteria.

**Table 5:** MIC and MBC of Dryopteris cristata AgNPs and aqueous extracts tested against Staphylococcus aureus and Escherichia coli The results demonstrate varying antimicrobial effectiveness, with *Dryopteris cristata* nanoparticles exhibiting inhibitory activity against *Staphylococcus aureus* at lower concentrations. However, both bacterial species were resistant to the aqueous extracts. MIC; minimum inhibitory concentration; MBC: minimum bactericidal concentration; AgNPs: silver nanoparticles

Organism	Antimicrobial agent	MIC	MBC
Staphylococcus aureus	*Dryopteris cristata* AgNPs	125μg	250 μg
	*Dryopteris cristata *aqeous extract	125μg	250 μg
Escherichia coli	*Dryopteris cristata* AgNPs	˃ 250μg	˃ 250 μg
	*Dryopteris cristata* aqeous extract	˃ 125μg	˃ 250 μg

Statistical analysis

The statistical results of the antimicrobial sensitivity tests for *D. cristata* aqueous extracts and DC-AgNPs against *S. aureus* and *E. coli* indicate varying degrees of effectiveness. The analysis was conducted using IBM SPSS Statistics for Windows, Version 20.0 (Released 2011; IBM Corp., Armonk, New York, United States).

Descriptive Statistics

The descriptive statistics reveal that *S. aureus* exhibited a mean sensitivity of 7.17 with a standard deviation of 1.15, suggesting a consistent response to the DC-AgNPs (Table 6). In contrast, *E. coli *showed no sensitivity, reflected by a mean of 0.00. This stark difference in mean sensitivity highlights the distinct responsiveness of the two bacterial strains to the treatment.

**Table 6:** Descriptive statistics The descriptive analysis tells the mean average value of the test result and the deviation (SD) from the mean

	N	Minimum	Maximum	Mean	Std. Deviation
Factor A	3	1.000	1.000	1.000	0.000
Staphylococcus aureus	3	6.500	8.500	7.167	1.155
Escherichia coli	3	.00	.00	0.000	0.000

Regression Analysis

Regression analysis was also carried out to determine the level of relationship between the concentration of reagents and resistance of the organism as well as model the relationship. The regression analysis demonstrates a strong correlation between the concentration of DC-AgNPs and the sensitivity of* S. aureus*, with an R value of 0.983 (Table 7). This indicates that approximately 96.7% of the variability in *S. aureus* sensitivity can be explained by the concentration of the nanoparticles, underscoring their potential effectiveness as an antimicrobial agent.

**Table 7:** Regression analysis R=0.983 indicates a relationship between the level of reaction as it touches *Staphylococcus aureus*.  R square means 96.7% of the data can be explained by the model/result.

Model	R	R Square	Adjusted R Square	Std. Error of the Estimate
1	0.983	0.967	0.958	0.112

ANOVA Results

The ANOVA results confirm a statistically significant difference between the two bacterial species, with an F value indicating significant effectiveness of DC-AgNPs against *S. aureus* compared to the resistance observed in *E. coli* (p < 0.001) (Table 8). This statistical significance reinforces the findings from the descriptive statistics and regression analysis.

**Table 8:** ANOVA This shows the overall significance of the test carried out. There is a statistically significant difference in the means of *Escherichia coli* and *Staphylococcus aureus*. One has mean of 0 and the other 7.167.

Model		Sum of Squares	df	Mean Square	F	Sig.
1	Regression Residual Total	1.450	1	1.450	115.562	0.001
		0.050	4	0.013		
		1.500	5			

Table 9 explains which of the variables (coefficient) contributes to the significance of the overall data.

**Table 9:** Coefficients

Model		Unstandardized Coefficients		Standardized Coefficients	t	Sig.
		B	Std. Error	Beta		
1	(Constant)	1.983	0.064		30.928	0.000
	Staphylococcus aureus	-0.135	0.013	-0.983	-10.750	0.001

## Discussion

The qualitative phytochemical analysis of *D. cristata* aqueous leaf extracts reveals a significant presence of several bioactive compounds, including tannins, flavonoids, glycosides, cardiac glycosides, and phenolics. Tannins are known for their astringent properties and potential antimicrobial activities [16]. The presence of glycosides, including cardiac glycosides, suggests potential cardiovascular benefits, as these compounds can influence heart function and may also contribute to antimicrobial activity by disrupting microbial cell membranes [17]. In contrast, the absence of terpenoids and saponins indicates a limitation in the range of bioactive compounds available for therapeutic applications, suggesting that the antimicrobial effects observed in this study may primarily stem from the other identified phytochemicals. Flavonoids possess strong antioxidant properties and have been documented to exhibit anti-inflammatory and antimicrobial effects [18].

These findings align with the research objectives focused on the antimicrobial activities of *D. cristata* in conjunction with AgNPs against *S. aureus* and *E. coli* [19,20]. The identified phytochemicals, particularly tannins, flavonoids, and phenolics, are well-documented for their antimicrobial properties, indicating that the aqueous extracts could significantly contribute to the observed antimicrobial effects. Phytochemicals such as tannins, flavonoids, and phenolics serve as capping agents that stabilize AgNPs, preventing their aggregation. This stabilization enhances the surface area of the nanoparticles, thereby improving their interaction with microbial cells.

AgNPs are known to bind to bacterial cell walls, which leads to disruption of the cell membrane. Phytochemicals can further enhance this disruption by weakening the cell wall or membrane through their own mechanisms, allowing AgNPs easier access to the cells. Additionally, AgNPs can stimulate the production of ROS, which causes damage to bacterial DNA, proteins, and lipids. Certain phytochemicals, particularly flavonoids, also generate ROS [19]. When combined with AgNPs, this effect is amplified, resulting in increased antimicrobial activity. Furthermore, AgNPs release Ag+ that are toxic to bacteria by interacting with essential cellular components like enzymes and proteins. Phytochemicals may aid in this ion release by lowering the energy barrier or increasing the availability of silver ions, thereby enhancing their antimicrobial effectiveness. Additionally, the combination of *D. cristata* extracts with AgNPs may enhance antimicrobial efficacy due to the synergistic effects of the phytochemicals and the nanoparticles [21]. This interaction could lead to improved bioactivity against resistant bacterial strains, warranting further investigation into the therapeutic potential of *D. cristata* in modern medicine.

Characterization of AgNPs synthesized using the aqueous extract of *D. cristata* was conducted through UV-Vis spectrophotometry and X-ray diffraction analysis, providing crucial insights into their formation, stability, and size [22]. The UV-Vis spectrophotometric analysis monitored the absorbance of the reaction mixture at various time intervals from zero to four hours, revealing a characteristic peak around 400 nm. This peak is indicative of SPR, a phenomenon that arises from the collective oscillation of conduction electrons in response to incident light. The absorbance values recorded at different wavelengths demonstrate the stability of the AgNPs over time, with the highest absorbance at 400 nm increasing from 1.270 to 1.384, confirming the successful synthesis of AgNPs. Such stability is essential for potential applications in biomedical fields, as it suggests that the nanoparticles can maintain their properties over time [23]. 

In conjunction with the UV-Vis analysis, the X-ray diffraction results indicated an average crystallite size of approximately 123.66 nm, which is relatively larger than typical nanoscale particles, which usually range from 1 nm to 100 nm [24]. These findings underscore the potential of DC-AgNPs for use in various applications, particularly in the development of antimicrobial agents against pathogens like *S. aureus* and *E. coli *[25]. The size of the nanoparticles is significant, as it influences their reactivity and potential biological applications. Larger nanoparticles may exhibit different properties compared to smaller ones, particularly in terms of antimicrobial efficacy [26]. The combination of UV-Vis spectrophotometry and X-ray diffraction analysis not only confirms the successful synthesis of AgNPs but also provides a comprehensive understanding of their optical properties and crystalline structure. Further investigations into the biological activity and mechanisms of action of these nanoparticles are warranted to fully explore their therapeutic potential. 

The FTIR analysis provided insights into the functional groups present and the potential interactions between the synthesized AgNPs and the plant extracts [27]. The presence of hydroxyl (O-H) and amine (N-H) functional groups, as indicated by the peaks at around 3400 cm−1, is critical for the stabilization and reduction of Ag+ during AgNP synthesis. The lower transmittance values at these wave numbers suggest strong interactions, likely due to the capping agents from the plant extracts. Assessment of antimicrobial efficacy of DC-AgNPs was assessed against two significant bacterial pathogens: *S. aureus* (ATCC 28923) and *E. coli* (ATCC 28922). The results from the antimicrobial susceptibility testing (AST), MIC, and MBC provide valuable insights into the effectiveness of these nanoparticles in combating bacterial infections (19). For *S. aureus*, the AgNPs exhibited potent antimicrobial activity, with the MIC indicating the lowest concentration at which the nanoparticles inhibited bacterial growth. This suggests that the AgNPs can effectively disrupt the cellular processes of this gram-positive bacterium, which is known for its resistance to various antibiotics [26].

In light of the findings presented in Table 4, it is evident that conventional antibiotics such as sparfloxacin and Augmentin exhibited significant inhibition zones against *S. aureus*, but the bacteria demonstrated resistance to Augmentin. This observation emphasizes the importance of exploring improved formulations of DC-AgNPs rather than implying that traditional antibiotics are inherently superior. The results underscore a critical need for further research focused on optimizing the properties of these nanoparticles, particularly by reducing their size or modifying synthesis methods. Such advancements could enhance the antimicrobial efficacy of DC-AgNPs, potentially enabling them to overcome bacterial resistance and serve as effective alternatives or complementary treatments for bacterial infections. By pursuing these avenues, we can contribute to the development of innovative strategies in the fight against antibiotic-resistant pathogens.

Limitations of the study

One significant limitation of this study is the size of the synthesized AgNPs, which had an average crystallite size of approximately 123.66 nm, larger than typical nanoscale particles ranging from 1 nm to 100 nm. This larger size may influence the reactivity and biological applications of the nanoparticles, potentially limiting their effectiveness compared to smaller nanoparticles. Additionally, the findings primarily focus on In vitro analyses, which do not account for the complexities of biological systems in vivo. Further research is necessary to validate the antimicrobial effects and therapeutic potential of *D. cristata* extracts and DC-AgNPs in living organisms. While the study suggests that the synergy between phytochemicals and AgNPs likely enhances antimicrobial efficacy, it does not thoroughly investigate the mechanisms of these synergistic effects. Understanding these interactions is essential for developing effective therapeutic applications and fully realizing the potential of *D. cristata* in modern medicine.

## Conclusions

The phytochemical analysis of *D. cristata* aqueous leaf extracts identified bioactive compounds such as flavonoids, glycosides, cardiac glycosides, tannins, and phenolics, which contribute to the antimicrobial effects observed against *S. aureus* and *E. coli*. The synthesis of AgNPs using the *D. cristata* aqueous extracts was confirmed by UV-Vis spectrophotometry and X-ray diffraction analysis, which demonstrated the successful formation, stability, and appropriate size of the nanoparticles. The antimicrobial susceptibility tests, along with the determination of the MIC and MBC, showcased the strong antimicrobial activity of the AgNPs against both gram-positive (*S. aureus*) and gram-negative (*E. coli*) bacteria. These results emphasize the potential of DC-AgNPs as alternative therapeutic agents in the fight against antibiotic-resistant infections, indicating their value in developing new antimicrobial approaches.
